# Supplementary material for: Investigation of the Associations between Diet Quality and Health-Related Quality of Life in a Sample of Swedish Adolescents
Source: Nutrients. 2022 Jun 15;14(12):2489. doi: 10.3390/nu14122489 (PMC9231196; doi:10.3390/nu14122489)
Supplement: Supplementary file 1 [file nutrients-14-02489-s001.zip › nutrients-1764907-supplementary.pdf]

Supplementary material

Table S1. Correlation matrix between SHEIA15, RADDS and KS-10

Variables	SHEIA15	RADDS	KS-10
SHEIA15	1	0.442 **	0.019
RADDS	0.442**	1	0.093 **
KS-10	0.019	0.093 **	1

** = $p < 0.01$

Table S2. Mean intake for SHEIA15 components and associated reference values based on national recommendations and guidelines.

	**Mean intake/day**	**Reference values/day**
	Gram/10MJ(SD)	Gram/10MJ
Wholegrain	33.3(40.0)	75[a]
	Gram/MJ(SD)	Gram/MJ
Fibre	2.08(0.73)	3.00[b]
	E%(SD)	E%
SFA	13.2(3.40)	<10[b]
MUFA	**13.3(3.54)**	10-20[b]
PUFA	4.65(1.49)	5-10[b]
Added sugar	10.7(8.10)	<10[b]
	Gram(SD)	Gram
Vegetables and fruit	208(175)	500[c]
Fish and shellfish	20.5(59.6)	45[d]
Red and processed meat	642.1(528.3)[e]	<500[b]

Values meeting reference values shown in bold. Abbreviations: Swedish Healthy Eating index for Adolescents 2015 (SHEIA15), mega joule (MJ), standard deviation (SD), energy percent (E%), saturated-fatty acid (SFA), monounsaturated-fatty acid (MUFA) and poly-unsaturated fatty acid (PUFA)

[a] Intake based on nutrient density in (44)
[b] Based on the (3)
[c] Based on Swedish food-based dietary guidelines: (26)
[d] Based on Swedish food-based dietary guidelines: (26) and advice from (Brugård Konde)
[e] Red and processed meat intake per week
